# [^89^Zr]Zr-DFO-TOC: a novel radiopharmaceutical for PET imaging of somatostatin receptor positive neuroendocrine tumors

**DOI:** 10.1186/s41181-024-00320-9

**Published:** 2024-12-18

**Authors:** Alexis M. Sanwick, Katherine N. Haugh, Evan J. Williams, Kala A. Perry, Nikki A. Thiele, Ivis F. Chaple

**Affiliations:** 1 Department of Nuclear Engineering, University of Tennessee, Knoxville, Knoxville, TN 37996 USA; 2 Chemical Sciences Division, Oak Ridge National Laboratory, Oak Ridge, TN 37831 USA

**Keywords:** Radiopharmaceuticals, Somatostatin receptors, Octreotide, Neuroendocrine tumors, Positron emission tomography

## Abstract

**Background:**

Neuroendocrine tumors (NETs) are clinically diverse types of tumors that can arise anywhere in the body. Previous studies have shown that somatostatin receptors (SSTRs) are overexpressed on NET cell membranes relative to healthy tissue, allowing for tumor targeting through radiolabeled somatostatin analogs (SSAs). This work aims to develop a novel ^89^Zr-labeled tracer incorporating the SSA, octreotide (TOC), for positron emission tomography (PET) imaging of SSTR + NETs and predictive dosimetry calculations, leveraging the excellent nuclear (t_½_ = 3.27 days, β+ = 22.3%, β^+^_avg_ = 395.5 keV) and chemical characteristics (+ 4 oxidation state, preferential coordination number of 7/8, favorable aqueous chemistry) of ^89^Zr. In combination with ^89^Zr, the known radiochemistry with the chelator deferoxamine (DFO) gives reason to believe that this radiopharmaceutical incorporating an octreotide conjugate will be successful in studying the suitability of detecting SSTR + NETs.

**Results:**

Radiochemical tracer assessment indicated that amounts as low as 0.1 nmol DFO-TOC can be effectively radiolabeled with ^89^Zr, while maintaining ≥ 95% radiochemical yield. The stability of the compound was found to maintain radiochemical yields of 89.6% and 88.7% on the benchtop and in mouse serum, respectively, after 9 days. Receptor binding and competitive receptor blocking assays compared AR42J (high SSTR expression), PC-3 (moderate SSTR expression), and PANC-1 (minimal SSTR expression) cell lines at time points up to 6 days. In vitro studies demonstrated highest uptake in AR42J cells, and statistically significant differences in tracer uptake were seen after 1 h. Internalization assays showed maximum internalization after 3 h for all cell lines.

**Conclusions:**

In this work, [^89^Zr]Zr-DFO-TOC was synthesized with radiochemical yields ≥ 95% and was found to remain stable in vitro at extended time points. In vitro cell studies demonstrated a statistically significant difference between receptor binding and blocking experiments. The development of this work shows potential to positively impact patient care through the predictive dosimetry calculations for the FDA-approved therapeutic agent [^177^Lu]Lu-DOTA-TATE, while allowing for imaging at extended timepoints and should be studied further.

## Introduction

Neuroendocrine tumors (NETs) are a heterogeneous group of malignancies that can originate anywhere in the diffuse neuroendocrine system but predominate in the gastroenteropancreatic tract (Cives and Strosberg 2014). NETs are characterized by a large range of histological appearances and biological behaviors, including the ability to synthesize and secrete a variety of biogenic amines and peptide hormones (Cives and Strosberg 2014). An estimated 12,000 people are diagnosed with NETs in the U.S. each year, with 1- and 5-year survival rates reaching 72.8% and 39.4%, respectively, emphasizing the need for early diagnosis (Man et al. 2018). The incidence and prevalence of the disease has steadily increased over the last 3 decades, potentially resulting from advancements in imaging, thereby leading to an improved and earlier diagnosis (Yao et al. 2008; Chauhan et al. 2020).

Significant progress has been made in recent years towards understanding the underlying biology and molecular pathways involved with NETs, particularly the role of somatostatin receptors (SSTRs) and their downstream pathways. SSTRs (SSTR1, 2 A/2B, 3, 4, and 5) belong to a family of G protein-coupled receptors with seven transmembrane-spanning proteins that bind the cyclic peptide somatostatin (SST) (Theodoropoulou and Stalla 2013). SST regulates cell growth and inhibits hormone secretion in the endocrine and exocrine systems (Brereton et al. 2015; Ampofo et al. 2020). Prior research has demonstrated that high SSTR expression is a feature of well-differentiated NETs, with SSTR2A presenting as the most commonly overexpressed receptor and SSTR1, SSTR3, and SSTR5 having a lower abundance (Mizutani et al. 2012). SSTR4 expression levels in NETs are often either notably lower than the other SSTRs or are not present (Mizutani et al. 2012). The characteristic expression of SSTRs present on NETs provides a valuable therapeutic target using somatostatin analogues (SSAs). Although SST demonstrates favorable characteristics and functions for therapeutic usage, the short bioactive half-life (< 1 min) and postinjection rebound of hormones pose significant issues for clinical use, highlighting the need for longer-lived SSAs to increase the pharmacological usability of the peptide (Brereton et al. 2015; Lamberts et al. 1996). As of 2024, there are three U.S. Food and Drug Administration (FDA) approved SSAs: octreotide (Sandostatin^®^), lanreotide (Somatuline^®^ Depot), and pasireotide (Signifor LAR^®^), each of which have a different binding affinity to the various SSTR subtypes (Gomes-Porras et al. 2020). This work focuses on the use of octreotide (TOC), which has a biological half-life of 1.5 to 2 h and demonstrates a high receptor affinity for SSTR2, therefore, inhibiting the proliferation of cells that express SSTR2 through activation of the tyrosine phosphate pathway (Buscail et al. 1994). The clinical use of radiolabeled SSAs for diagnosis and treatment of neuroendocrine tumors has shown great success with the FDA-approval of five SSTR-targeting radiopharmaceuticals including the imaging agents [^111^In]In-pentetreotide, [^64^Cu]Cu-DOTA-TATE, [^68^Ga]Ga-DOTA-TATE, [^68^Ga]Ga-DOTA-TOC, and the therapeutic agent [^177^Lu]Lu-DOTA-TATE (Hennrich and Benešová 2020; Hennrich and Kopka 2019). However, from a dosimetry perspective, limitations arise pertaining to the short half-lives of ^68^Ga and ^64^Cu, 68 min and 12.7 h, respectively. Studies have shown that the absorbed dose of [^177^Lu]Lu-DOTA-TATE cannot be predicted from a single [^68^Ga]Ga-DOTA-TOC PET scan and leads to a predicted absorbed dose that is highly overestimated (Bruvoll et al. 2023). This highlights the dire need for a matched pair imaging agent that can provide a closer predictive dosimetry for [^177^Lu]Lu-DOTA-TATE, for which, in this work we propose to develop a novel ^89^Zr-labeled TOC analogue for positron emission tomography (PET) imaging of SSTR positive (SSTR+) NETs.

Zirconium-89 (^89^Zr) is a promising radiometal with favorable nuclear characteristics (t_1/2_ = 78.41 hours, ${\beta ^ + }$= 23%, $\beta _{avg}^ +$ = 396.9 keV) for applications in PET (Queern et al. 2017). ^89^Zr has a longer half-life than the currently employed radiometals in SSTR PET imaging, including ^64^Cu and ^68^Ga, allowing for imaging at extended timepoints, while presenting with a positron branching ratio that is acceptable for imaging. The increased imaging timepoints could allow for favorable predictive dosimetry calculations that are necessary for the FDA approved counterpart, [^177^Lu]Lu-DOTA-TATE. ^89^Zr can be readily produced by a cyclotron using a naturally abundant ^89^Y target resulting in high specific activity ^89^Zr and is widely available and affordable (Queern et al. 2017). In addition to its nuclear characteristics, ^89^Zr exhibits excellent chemical characteristics (+ 4 oxidation state, preferential coordination number of 7 or 8, favorable chelation chemistry), all of which are desirable for the development of radiopharmaceuticals that employ chelators that meet these chemical requirements (Deri et al. 2013). The chelation chemistry of ^89^Zr has been widely explored with deferoxamine (DFO) (the most commonly used chelator) which is composed of three hydroxamate moieties that chelate ^89^Zr (Imura et al. 2021; Holland et al. 2010).

**Fig. 1:**
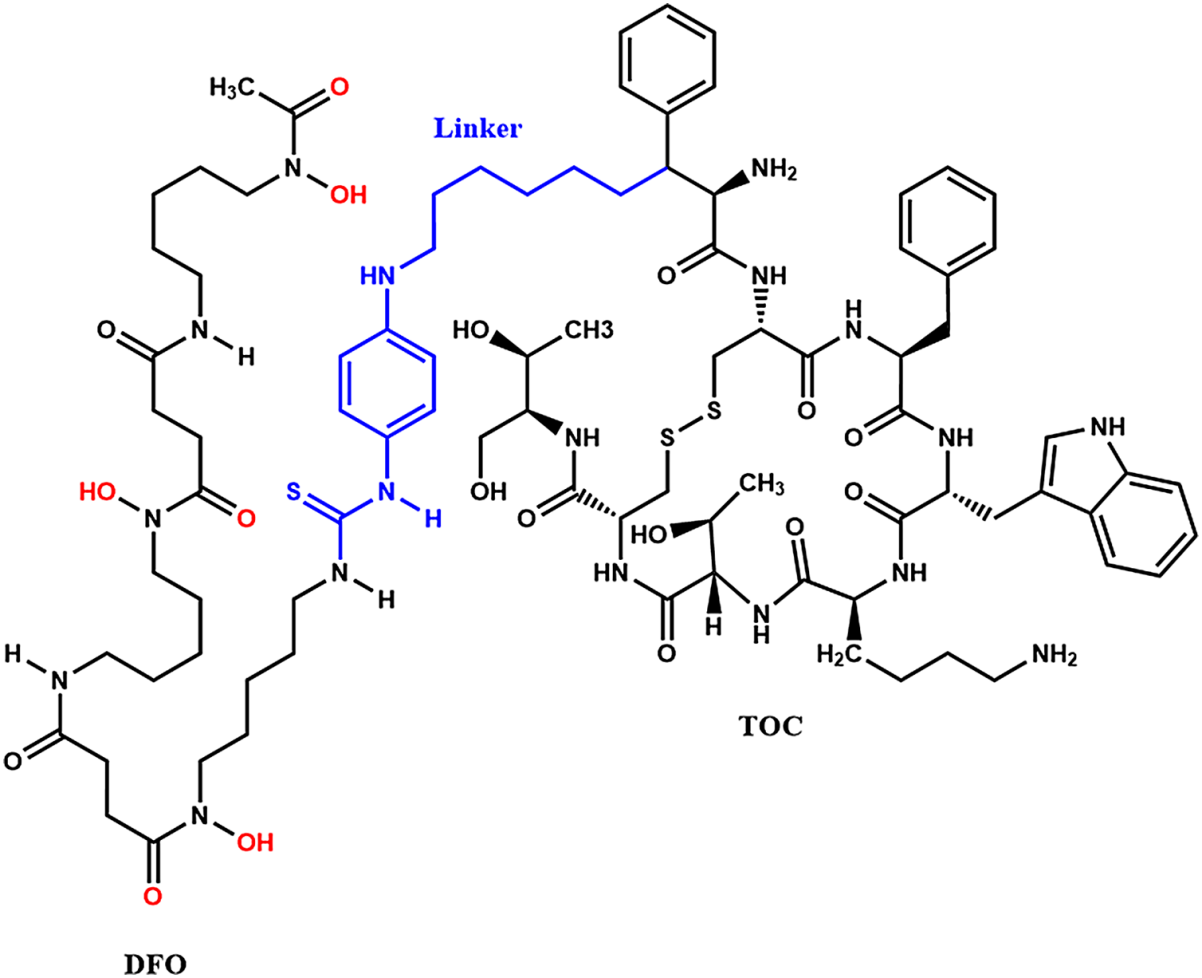
Chemical structure of DFO-TOC

In this work we report for the first time, to our knowledge, in vitro data using a novel [^89^Zr]Zr-DFO-TOC radiopharmaceutical, shown in Fig. 1. The uptake of [^89^Zr]Zr-DFO-TOC using AR42J cells as a high SSTR expressor, PC-3 cells as a moderate SSTR expressor, and PANC-1 cells as a minimal SSTR expressor were explored at various time points and under varying conditions. This study demonstrates the potential use of ^89^Zr-labeled TOC radiopharmaceuticals for targeting SSTR2 + cell lines in vitro and illustrates the need for future dosimetry calculations derived from in vivo studies using this radiopharmaceutical.

## Methods and materials

### Reagents

All reagents were purchased from Fisher Scientific (Hampton, NH) unless otherwise noted. [^89^Zr]Zr-oxalate (1 M oxalic acid, radionuclidic purity > 99.99%) was purchased from the University of Alabama at Birmingham Cyclotron Facility. DFO-TOC (Deferoxamine-C6-Dphe-Cys-Tyr-DTrp-Lys-Thr-Cys-Thr-ol, peptide purity = 92.9%, molecular weight = 1901.3 g/mol) was purchased from CPC Scientific (San Jose, CA).

All cell lines and cell media were purchased from the American Type Culture Collection (ATCC) organization (Manassas, Va). PANC-1 cell lines were cultured using Dulbecco’s Modified Eagle’s Medium with 10–20% fetal bovine serum (FBS) and 0.1% gentamycin. PC-3 and AR42J cell lines were cultured using Kaighn’s Modification of Ham’s F-12 Medium with 10–20% FBS and 0.1% gentamycin.

### Radiochemical yield determination

^89^Zr was produced as previously described and obtained in the form of [^89^Zr]Zr-oxalate (Queern et al. 2017). To neutralize [^89^Zr]Zr-oxalate, an equal volume of [^89^Zr]Zr-oxalate and 0.5 M Thermo Scientific HEPES (concentration 99%) buffer were mixed. The pH of the sample was then adjusted using 5.0 M NaOH. For all radiolabeling experiments, 100 µCi (3.7 MBq) of ^89^Zr (pH ~ 7.4) was incubated with varying molar quantities of DFO-TOC and 1.0 M HEPES buffer in a final incubation volume of 200 µL. The reaction was incubated on a thermomixer at 37 °C and 800 rpm for 30 min. The molar activity was determined by radiolabeling 1 nmol of DFO-TOC with varying activities of ^89^Zr up to 1500 µCi (55.5 MBq) and 1.0 M HEPES buffer in a final incubation volume of 200 µL. Samples were incubated using the same conditions as the radiochemical yield assays. Samples were analyzed using an Agilent (Santa Clara, CA) 1260 Infinity II high-performance liquid chromatography (HPLC) instrument with a LabLogic (Chantilly, VA) Flow-RAM radio-HPLC detector. Radiochemical yields were determined via radio-HPLC using a reversed-phase C18 selectivity column (5 μm particle size, 150 mm length, and 4.6 mm diameter). The gradient method was as follows (A = 0.1% TFA in water, B = 0.1% TFA in acetonitrile): 100% A from 0 to 2 min, linearly increased to 100% B from 2 to 4 min, 100% B from 4 to 12 min, linearly decreased to 100% A from 12 to 14 min, and 100% A from 14 to 15 min.

### Benchtop and mouse serum stability studies

The samples were assessed for benchtop stability and stability in mouse serum only if a radiochemical yield of 95% or greater was achieved. Benchtop stability was assessed at time points of 30 min, 1 h, 1 day, 2 days, 3 days, 6 days, and 9 days using an Eckert & Ziegler (Valencia, CA) AR-2000 instant Thin Layer Chromatography (iTLC) and radio-HPLC. Diethylenetriamine pentaacetic acid was used as the mobile phase for iTLC, while radio-HPLC used the gradient described previously. Stability in mouse serum was assessed by adding 100 µL of a 200 $\:\mu\:$Ci (7.4 MBq) [^89^Zr]Zr-DFO-TOC sample to 400 µL of prefiltered mouse serum. No further sample purification was conducted before injection on the radio-HPLC. All samples remained on a thermomixer at 37 °C and 800 rpm, and aliquots were removed for analysis by using iTLC and radio-HPLC, after 1 h, 1 day, 2 days, 3 days, 5 days, 6 days, and 9 days.

### In vitro assays with AR42J, PC-3, and PANC-1 cells

#### Binding assays

Cells were seeded in Fisherbrand™ 12-well surface-treated culture plates with 1 million cells per well for each cell line and were allowed to adhere overnight under normal cell culture conditions. Samples were not purified to remove excess DFO-TOC. For receptor binding and blocking experiments, each well was incubated with [^89^Zr]Zr-DFO-TOC (0.025 nmol DFO-TOC per well, 1 µCi (0.037 MBq) of [^89^Zr]) in 1mL of cell media. For receptor blocking experiments,

2.5 nmol unlabeled DFO-TOC was added to each well, in addition to [^89^Zr]Zr-DFO-TOC. Culture plates were incubated with the tracer for 1 h, 3 h, 1 day, 3 days, 5 days, and 6 days. At each timepoint, the media was removed and the cells were washed with PBS twice. Cells were detached using 1 mL of 1 M NaOH and collected in a microcentrifuge tube for analysis using a Revvity (Waltham, MA) Wizard2 Gamma Counter. BCA assays were used to estimate protein concentrations per the manufacturer’s instructions.

#### Internalization assays

Cells were seeded in Fisherbrand™ 12-well surface-treated culture plates with 1 million cells per well for each cell line and were allowed to grow overnight under cell culture conditions. For internalization experiments, each well received 0.025 nmol DFO-TOC radiolabeled with 1 µCi (0.037 MBq) of [^89^Zr]Zr-DFO-TOC in 1 mL of cell media. Plates were incubated with [^89^Zr]Zr-DFO-TOC for 30 min, 1 h, and 3 h. Cells were detached from the wells using 800 µL of 0.05% Gibco trypsin incubated for 5 min. Cells were sedimented using a benchtop centrifuge at 7000 g for 5 min. The supernatant was removed, and the cells were resuspended in 200 µL of 0.1 M Supelco sodium citrate (pH 2) for 5 min. The cells were sedimented using a benchtop microcentrifuge and the supernatant was added to the trypsin. The supernatant and cell pellet activities were determined using the Wizard2 Gamma Counter.

### Flow cytometry

Flow cytometry was performed on AR42J, PC-3, and PANC-1 cell lines to determine SSTR expression. To determine viability, cells were stained with 5–7 µL BioLegend (San Diego, CA) 7-AAD viability solution per 1 million cells in 500 µL BioLegend cell staining buffer. Viability samples were incubated for 10 min at 4 °C. Cells were stained for SSTR expression using 5 µL of BD Biosciences (Franklin Lakes, NJ) Somatostatin Alexa Fluor^®^ 488 Mouse Anti-Human fluorophore per million cells. Cell staining buffer was used to make the final sample volume 100 µL. Cells were incubated for 45 min at 4 °C. Sample staining and gating were determined using a Cytek^®^ (Fremont, CA) Northern Lights Flow Cytometer and analyzed using Cytek^®^ SpectroFlo software.

### Data analysis and statistical analysis

All data was analyzed using GraphPad Prism (San Diego, CA) and is presented as a mean ± standard deviation. Statistical analysis was completed using a paired nonparametric t-test (α = 0.05) to compare cell total binding and cell blocking groups.

## Results

### Chemical assessment of [89Zr]Zr-DFO-TOC

**Fig. 2:**
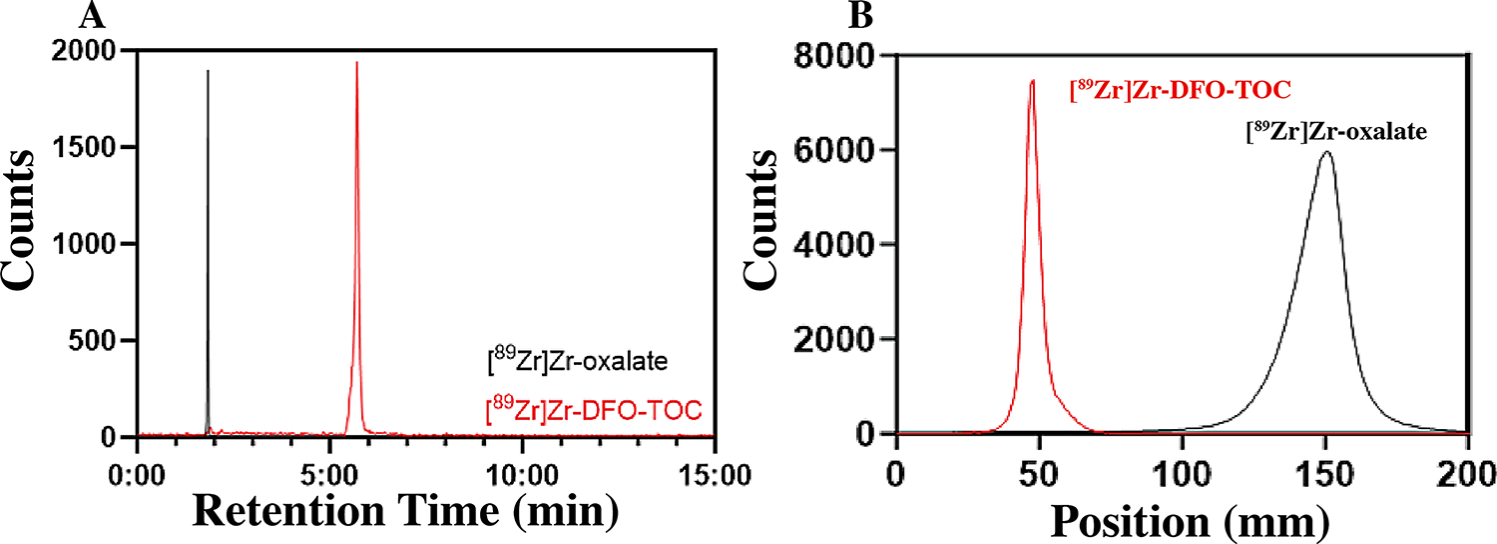
Comparison of retention times of [^89^Zr]Zr-oxalate and [^89^Zr]Zr-DFO-TOC by radio-HPLC (**A**) and iTLC (**B**)

**Fig. 3:**
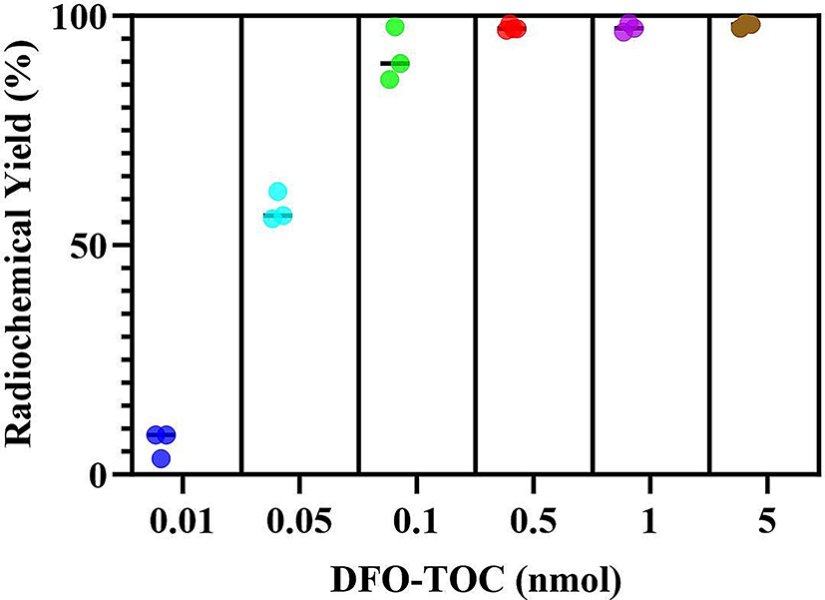
Radiochemical yields of [^89^Zr]Zr-DFO-TOC determined via radio-HPLC. Samples were prepared to contain 100 µCi of [^89^Zr]Zr-oxalate (pH. = 7.4), varying amounts of DFO-TOC, and 0.5 M HEPES buffer in a final volume of 200 µL. Samples were incubated for 30 min at 37 °C and 800 rpm prior to analysis

The samples were analyzed for radiochemical yield and stability using iTLC and radio-HPLC where representative spectra are shown in Fig. 2. As seen from the HPLC chromatogram in Fig. 2A, free ^89^Zr was eluted from the column around 2 min, whereas the [^89^Zr]Zr-DFO-TOC complex had a retention time of 6 min. The iTLC chromatogram is shown in Fig. 2B, in which the peak at 50 mm represents [^89^Zr]Zr-DFO-TOC, whereas the peak found around 130 mm corresponds to free ^89^Zr. The radiochemical yields for varying amounts of DFO-TOC are plotted in Fig. 3. The results of this study demonstrate that ^89^Zr is effective in radiolabeling as low as 0.1 nmol DFO-TOC while maintaining greater than 95% radiochemical yield. Molar activity was determined to be greater than 1.5 mCi/nmol (55.5 MBq/nmol).

### Benchtop and mouse serum stability

**Fig. 4:**
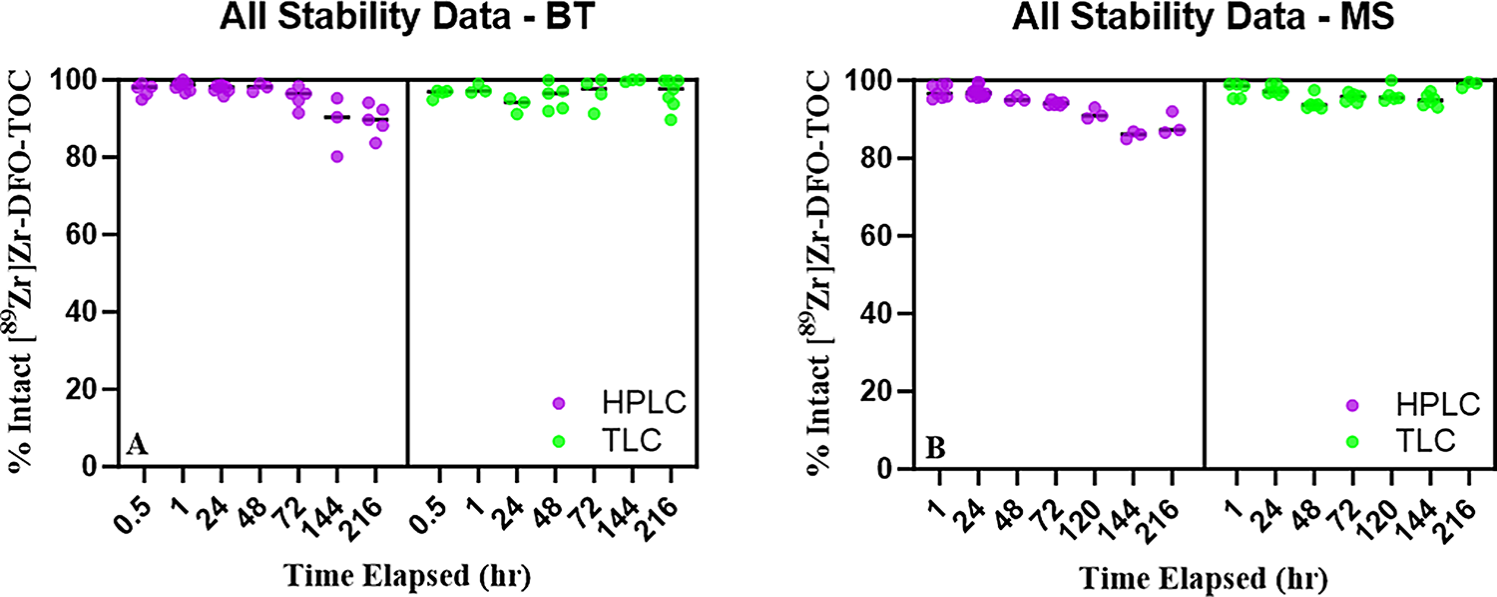
Stability of [^89^Zr]Zr-DFO-TOC on the benchtop (**A**) and in mouse serum (**B**) at timepoints up to 9 days determined via HPLC (purple) and iTLC (green)

The radiochemical yields of the benchtop samples are shown in Fig. 4A. and the mouse serum samples in Fig. 4B. Assessment of the radiotracer stability on the benchtop resulted in radiochemical yields of 90 ± 4% after 9 days using radio-HPLC. On iTLC, the radiochemical yield on benchtop was 97 ± 4%. Similarly, in mouse serum after 9 days, the radiotracer was determined to have yields on the radio-HPLC and iTLC of 89 ± 2% and 99 ± 1%, respectively.

### In vitro assays

**Fig. 5:**
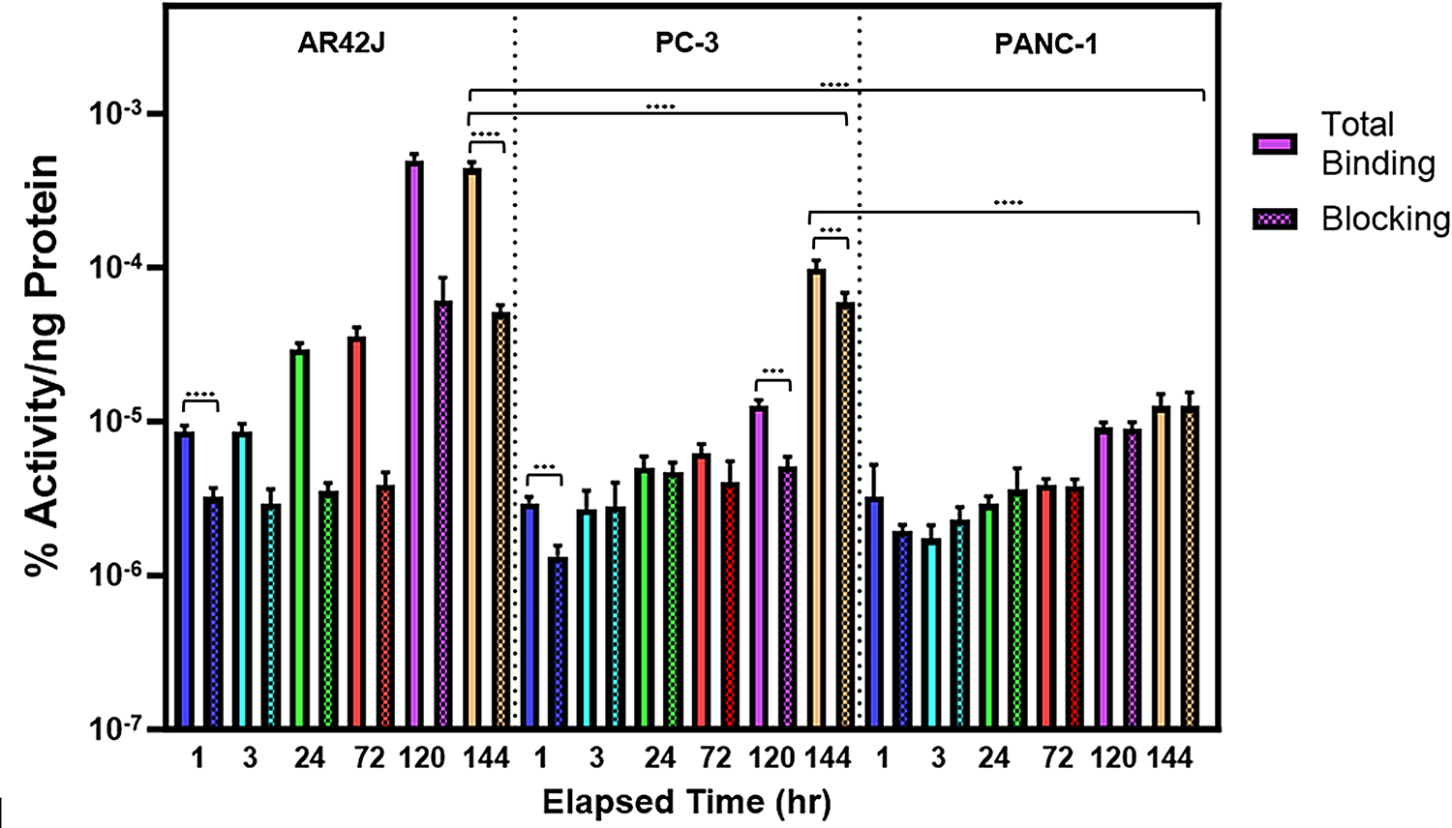
Receptor binding assays using AR42J, PC-3, and PANC-1 cell lines at time points up to 6 days. Radiotracer specificity was assessed by comparing the total binding of the tracer (left) to the tracer binding in the presence of 100x excess unlabeled DFO-TOC (right)

The tracer radiochemical purity was found to be > 95% via radio-HPLC before in vitro assays were conducted. The specificity of [^89^Zr]Zr-DFO-TOC for SSTR was assessed using AR42J (SSTR2 high expression), PC-3 (SSTR2 moderate expression), and PANC-1 (SSTR2 minimal expression) cell lines. The results of the binding and blocking assays are plotted in Fig. 5. AR42J was found to have a higher uptake than PC-3 and PANC-1 cell lines at all time points. There was a statistically significant difference between AR42J binding versus PC-3 and PANC-1 cell binding after 1 h (*P* < 0.0001 comparing AR42J to PC-3 and *p* = 0.0001 comparing AR42J to PANC-1). AR42J cell binding reached saturation after 5 days. Cell blocking studies used unlabeled DFO-TOC as a blocking agent to assess receptor affinity. The AR42J cell line demonstrated a statistically significant difference between the blocking and total binding groups after 1 h (*p* < 0.0001) and the PC-3 cell line at 120 h (*p* = 0.0001). No statistically significant difference was observed between binding and blocking groups for the PANC-1 cell line at all time points.

### Internalization studies

**Fig. 6:**
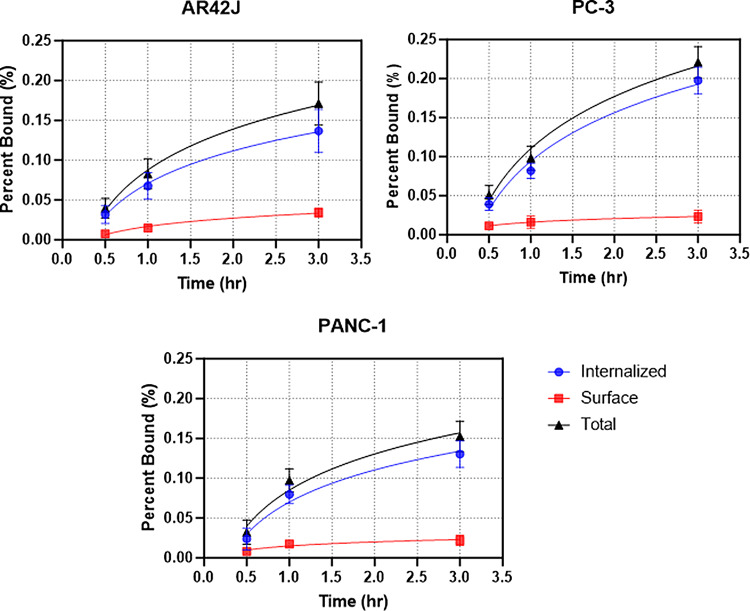
Internalization assay in AR42J, PC-3, and PANC-1 cell lines at timepoints up to 3 h

Cell internalization experiments were carried out to determine kinetics of the radiotracer uptake in all three cell lines, and the results are shown in Fig. 6. All cell lines reached maximum cell internalization after 3 h. A higher percentage of the tracer was internalized in the PC-3 cell line when compared to AR42J and PANC-1 cell lines.

### Flow cytometry

**Fig. 7:**
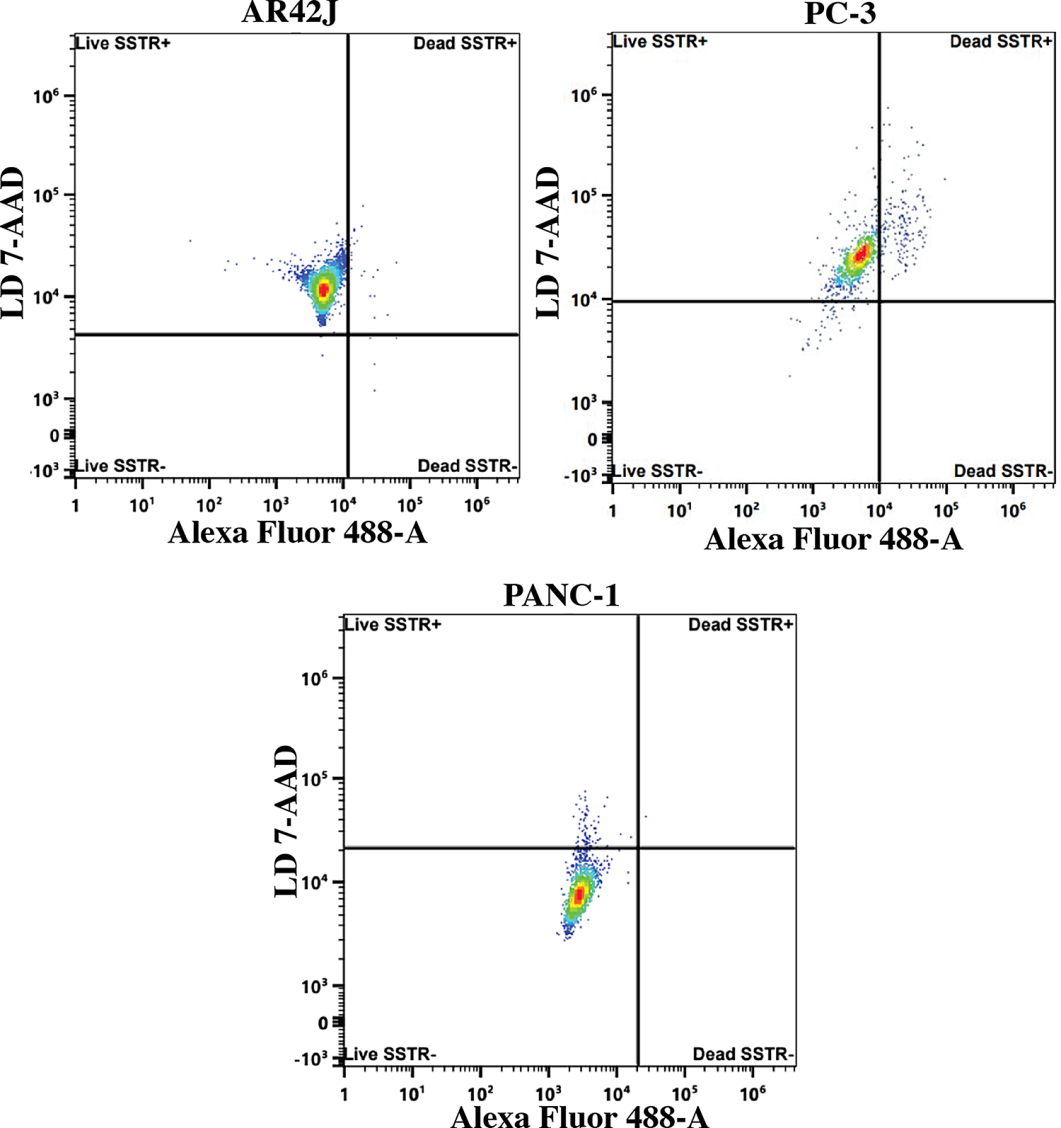
Flow cytometry analysis of AR42J, PC-3, and PANC-1 cell lines for determination of the SSTR expression

Flow cytometric analysis was conducted using human somatostatin-transfected cells stained with Alexa Fluor 488 Mouse Anti-Human Somatostatin. SSTR expression was investigated using AR42J, PC-3, and PANC-1 cell lines and was determined to be 99.31%, 80.30%, and 2.80%, respectively. These results are shown in Fig. 7.

## Discussion

TOC is the first FDA-approved SSA to be implemented in the management and treatment of NETs and has led to current FDA-approved SSTR-targeting radiopharmaceuticals such as [^68^Ga]Ga-DOTA-TOC. Although [^68^Ga]Ga-DOTA-TOC has demonstrated clinical success, its use is limited by the short half-life of ^68^Ga ($\:{\text{t}}_{1/2}=68\:\text{m}\text{i}\text{n})$. This limitation can be fulfilled by labeling TOC with a longer-lived radionuclide, such as ^89^Zr ($\:{\text{t}}_{1/2}=78.4\:h)$, which would allow for PET imaging at longer timepoints. Our studies aimed to determine the feasibility of a ^89^Zr-labeled TOC tracer for PET imaging of SSTR+ NETs based on radiochemical yield, stability, and in vitro studies. Despite past interest in the development of [^89^Zr]Zr-DFO-TOC, our work represents the first study of the in vitro characteristics of this radiopharmaceutical agent (Wadas et al. 2020; Noor et al. 2021). [^89^Zr]Zr-DFO-TOC was synthesized under mild conditions with high radiochemical yield (≥95%), demonstrating both high affinity for ^89^Zr for DFO (which has been previously confirmed in the literature), and that conjugation of TOC to DFO did not affect the radiochemistry of the radiometal to the chelator. A comparison of sample yields acquired from HPLC and iTLC demonstrated slightly lower yields on HPLC, which can be attributed to the higher detector sensitivity of HPLC versus iTLC methods, as supported by previously published work (Hooijman et al. 2022). The high stability observed for the samples in mouse serum supports our hypothesis that minimal ^89^Zr transchelation to proteins will occur in vivo, although further studies are warranted. With TOC having a biological half-life of 90 min, more than 10 biological half-lives will have passed before the stability decreases to ≤ 95% on benchtop and in mouse serum, although further studies are warranted to assess the biological stability of DFO-TOC. In vitro binding assays revealed a high specificity of the radiotracer for SSTR, which was consistent with the relative SSTR expressions of AR42J, PC-3, and PANC-1 cell lines determined using flow cytometry and also validated by previously published studies in the literature (Fottner et al. 2010; Sun et al. 2011). Although chelator and radiometal modifications are shown to affect affinity of the ligand to the SSTR, the in vitro receptor binding of [^89^Zr]Zr-DFO-TOC to SSTR has potential to demonstrate similar binding affinity and characteristics to FDA-approved [^68^Ga]Ga-DOTA-TOC and should be studied in vivo. TOC demonstrates receptor affinity for SSTR2 and SSTR5, which agrees with the high AR42J (> 70% SSTR2/<20% SSTR5) and low PANC-1(barely detectable SSTR2/SSTR5) uptake due to the receptor expression (Lamberts et al. 1996; Fottner et al. 2010). Low [^89^Zr]Zr-DFO-TOC uptake occurs in PANC-1 due to the low SSTR2 expression (Fottner et al. 2010). SSTR is hypothesized to have fast receptor internalization kinetics, potentially corresponding to the low internalization percentage seen in these experiments, and further work should be conducted to validate this hypothesis. In the case of AR42J, SSTR2 internalization of [Tyr3, Thr8]-octreotide has been observed as early as 2.5 min, though it is important to note that changing the polarity of the peptide can lead to variations in percent internalization (Vaidyanathan et al. 2003; Hofland et al. 1999). Additionally, the low SSTR2 expression found in PC-3 and PANC-1 cell lines, and fast SSTR2 internalization kinetics support the low internalization rate seen in these studies (Sun et al. 2011; Tatoud et al. 1995). Further experiments at earlier timepoints are warranted to draw concise conclusions regarding the [^89^Zr]Zr-DFO-TOC internalization of these cell lines and to fill the gap of TOC internalization kinetics missing in the literature.

## Conclusion

This work demonstrated the successful synthesis and in vitro characterization of a novel [^89^Zr]Zr-DFO-TOC radiotracer for in vitro targeting of SSTR in three different cell lines. The results of these studies support that neither the conjugation of DFO to TOC, or radiolabeling DFO with ^89^Zr, affect the radiochemistry or in vitro binding affinity of TOC to SSTR. The ease of synthesis, stability, and in vitro specificity to SSTR warrant future in vivo studies incorporating PET imaging of mice bearing SSTR + xenograft tumors. The 3.3-day half-life of ^89^Zr can be leveraged to provide more accurate information related to the predictive dosimetry pertinent for future theranostic applications with the long-lived therapeutic radiopharmaceutical, [^177^Lu]Lu-DOTA-TATE (t_½_ = 6.6 days).

## Data Availability

Data is available upon reasonable request to the corresponding author.

## Abbreviations

NETs: Neuroendocrine tumors
SSTRs: Somatostatin receptors
SST: Somatostatin
TOC: Octreotide
PET: Positron emission tomography
SSTR+: Somatostatin receptor positive
HPLC: High-performance liquid chromatography
iTLC: Instant thin layer chromatography
